# Swedish consumers´ attitudes and values to genetic modification and conventional plant breeding – The case of fruit and vegetables

**DOI:** 10.1080/21645698.2021.1921544

**Published:** 2021-05-10

**Authors:** Sara Spendrup, Dennis Eriksson, Fredrik Fernqvist

**Affiliations:** a Department of People and Society, Swedish University of Agricultural Sciences, Alnarp, Sweden; b Department of Plant Breeding, Swedish University of Agricultural Sciences, Alnarp, Sweden

**Keywords:** Attitudes, schwartz value theory, gmo, conventional plant breeding, fruit and vegetables, consumers

## Abstract

This study examined public attitudes to genetic modification (GM) and conventional plant breeding and explored general differences in attitudes to these two types of breeding concepts, including the effect of individual personal characteristics such as gender and age. It also sought to identify the influence of personal values linked to attitudes to GM crops and conventional plant breeding, following Schwartz value theory. Relations between specific values and attitudes to GM organisms (GMOs) have been studied previously, but not gender- and age-specific relations between specific values and attitudes to conventional plant breeding. Data were collected in this study using a questionnaire completed on-line by 1500 Swedish consumers in 2019. The questionnaire covered three different aspects: 1) sociodemographic data, including gender and age; 2) attitudes to GMO/conventional plant breeding; and 3) values, measured using the human values scale. It was found that consumers expressed more positive attitudes to conventional plant breeding than to GMO, men expressed more positive attitudes to both conventional plant breeding and GMO than women did, and younger consumers expressed more positive attitudes to GMO than older consumers did. A negative correlation between attitudes to conventional plant breeding and the value ‘tradition’, but no correlation to ‘universalism’, ‘benevolence’, ‘power’ or ‘achievement’, was identified for men. For women, correlations between attitudes to conventional plant breeding and ‘benevolence’ (neg.) and ‘achievement’ (pos.) were found. For both men and women, attitudes to GMO were negatively influenced by ‘universalism’ and ‘benevolence’, and positively influenced by ‘power’ and ‘achievement’. The implications of these results are discussed.

## 1. Introduction

Use of genetic modification (GM) in plant breeding is a contentious issue. Those in favor of this technology cite the ease and efficiency of developing improved plant resistance to diseases and pests, increased tolerance to abiotic stresses such as rain or drought and improved consumer-oriented quality characteristics such as taste, appearance, shelf-life and nutritional content .^1^^,2,3,4,5,6,7,8^ Those opposing GM technology cite concerns and potential risks, such as negative changes in nutritional qualities, increasing allergenic substances, internal production of insect toxins in crops, and unintentional gene transfer into wild populations and associated potential negative effects on natural ecosystems.^9–11^ However, food systems are facing multiple challenges, such as issues related to climate change, food security,^12–14^ and increased pest and phytosanitary problems.^15^ In light of these challenges, scientific progress in plant breeding is likely to play an important, if not necessary, role for developing future crops.

Much research has already been devoted to consumer resistance to GMO, which has resulted in a well-grounded understanding of explanatory variables for why consumers express negative attitudes to GM food ^16,17,18^ Concerns among European consumers about GM foods mainly relate to perceived absence of benefits, concerns about safety and a perception that the products are ‘unnatural’.^19,20^ GMO products may be perceived as representing new and different food products, and it has been found that consumers may express food neophobia regarding these products.^21^ This in turn contributes to a negative relationship and lack of acceptance of GM foods.^22^ Food neophobia and reluctance to try unfamiliar food products have been widely associated with GM avoidance.^23–26^ There are also concerns about equality, as consumers believe it is negative for a few companies to dominate the market.^27^ Some studies have reported that the perceived high risk of GM foods leads to reduced acceptance, negative attitudes and negative emotional responses.^16,28–31^

However, while consumer resistance to GM food still exists, longitudinal studies such as the Eurobarometer show that this resistance is not as entrenched as it was previously. Between 2006 and 2019, the level of concern about presence of GM ingredients in food or drinks declined from over 60%^32,33^ to 27%,^19^ illustrating a significant decrease in the level of concern among EU consumers. Studies examining incentives for consumers to buy GM foods show that positive attitudes increase when advantages relating to climate change and ensuring food security are highlighted.^34^ Additionally, health and nutrient benefits (e.g. vitamins),^6^ and reduced price and perceived quality,^35,36^ have been proven to have positive effects on consumer willingness to buy GM foods.

Yet, given the prevailing political situation and mistrust of GM crops among up to one-third of European consumers,^17,19^ it is reasonable to assume that, for the foreseeable future, only crops deriving from conventional plant breeding methods will reach the market in Europe. Although much GM research on horticultural crops is taking place in Europe, very few applications have been submitted for commercial approval. The GM products that have received market authorization are nearly all imported livestock feeds, and it is practically impossible to receive authorization for cultivation of a GM crop in the European Union (EU). One obstacle is that an application never gets support from a qualified majority of EU member states, for reasons that are mostly of a political nature. A prevalent argument is that member states display voting behavior that reflects the opinion of their citizens. This may be true to some extent, but according to the latest Eurobarometer report the concern about GM crops among EU consumers has decreased significantly.^19^ For a better understanding of the political and other motives behind the regulatory gridlock facing GM crops in the EU, it is important to understand the factors that may influence public opinion on this type of technology.

This study compared two types of plant breeding concepts (GM technology and conventional plant breeding) with regard to consumer attitudes and values, following Schwartz value theory.^37^ Specific objectives of the study were: i) to determine consumer attitudes in general and differences between gender and/or different age groups, regarding GM crops and conventionally bred crops; and ii) to explore and identify value structures (in accordance with Schwartz value theory) linked to consumer attitudes to GM crops and conventionally bred crops.

Values and attitudes have been identified as principal explanatory components for the psychological mechanism/s causing people to behave differently or make different choices. Values, attitudes and behaviors are suggested to operate in a hierarchical structure, where values represent the most profound level, the starting point for developing attitudes, which in turn lead to a certain behavior.^23,38,39^ The reasons why a person perceives an object positively or negatively can thus be identified by measuring attitude in relation to the object. In the present study, attitude was operationalized according to the definition by Eagly & Chaiken [,^40^ p. 1) as “a psychological tendency expressed through assessing an object with some degree of favor or disfavor”. Values can be seen as goals that provide a general orientation and organization in life^41^ and guide consumer daily life.^42^ Many previous studies have explored links between values and food, e.g. following a vegetarian diet,^43^ influencing pro-environmental behavior^44^ and ethical and sustainable consumption patterns.^39,45–48^ Examination of the value structure behind attitudes to GM technology and conventional plant breeding can increase understanding of the origins of the attitudes expressed.

Conventional breeding is commonly referred to in the literature as the development of new cultivars using conventional tools and natural processes for manipulating the plant genome within the natural genetic boundaries of the species, as opposed to molecular plant breeding which may go beyond these boundaries.^49^ However, some conventional technologies, such as tissue culture-based wide crosses and bridge crosses, transgress the boundaries of natural processes. Most technologies based on the science of genetics and developed from the early 20^th^ century until the onset of molecular biology methods in the 1970s are considered conventional breeding. Breeding of horticultural crops is now carried out using both conventional and molecular methods. Heterosis was exploited commercially early on in hybrid breeding of e.g. onion,^50^ and hybridization and selection has been applied successfully in a number of other crops, such as strawberry, apple, tomato and squash.^51^ Hybridization of watermelon parental lines with different chromosome numbers has been employed to generate seedless fruit. In *Citrus* species, somatic hybridizations via protoplast fusion and also cybrids (artificial hybrid cells) have been commonly used to generate new cultivars, including seedless cultivars.^52^

Transgenic, or GM, technology has also been applied extensively in horticultural crops. Dias and Ortiz^53,54^ reviewed the advances in multiple crops, including tomato, eggplant, squash, potato, cucurbits, brassicas, lettuce, alliums, carrots, papaya, plum and banana. They concluded that, due to fast cultivar turnover and high GM deregulation costs, investment in transgenic breeding of horticultural crops remains rather low.^54^ Resistance in papaya to ringspot virus is an example of a commercial success resulting from GM technology.^55^ GM-engineered resistance to counter the threat of fusarium wilt disease in banana also shows great promise,^56^ while citrus cultivation stands to benefit from GM trait management, particularly for disease resistance.^57^ High potential of genome editing, the latest development in plant breeding technologies, has also been highlighted for horticultural crops.^58–60^ This technology has already found exciting applications, such as *de novo* domestication of wild tomato.^61,62^

Addressing actual breeding techniques is important, as consumers are becoming increasingly interested in these aspects of their consumption choices.^63,64^ It is likely that consumers in general have little understanding of all the breeding technologies (including conventional) that have been developed since the early 20^th^ century and used in production of the majority of all food products consumed today. The distinction between conventional and GM is likely understood as relating to the “naturalness” of genetic changes or combinations. This study focused on breeding of fruit and vegetables, a less-studied product category as regards consumer food acceptance of GM products. Considering the urgent need to develop plant material to meet future challenges, increased understanding of consumer attitudes and linked values toward available techniques can provide researchers and industry with a deeper understanding of the values guiding consumers in [non)-acceptance of innovations achieved through the use of GM technology or conventional plant breeding.

## 2. Values and Attitudes to GMO and Conventional Plant Breeding

A large number of studies have examined consumer attitudes to GM foods, but fewer studies have explored corresponding attitudes to conventional plant breeding, probably because GM food historically has been perceived as controversial, while conventional plant breeding is not. In a study comparing consumer attitudes, van den Heuvel et al. ^65^found that respondents preferred conventionally bred products over GMO, mainly due to natural breeding practice, good sensory appeal and low concerns among consumers about this breeding practice. Similar findings were made by Tanaka,^66^ with consumers expressing more positive attitudes to conventionally bred plants compared with GM crops. A general preference among consumers for conventionally bred plants over GM crops was also identified by Lampila et al.,^67^ who explored consumer perceptions of appropriateness and acceptability and found that consumers perceived conventional breeding methods as being more appropriate and acceptable. As discussed by Jaeger et al.,^68^ the name of the technology may also influence the associations consumers make. A novel technology such as GMO may be associated with risk, while conventional plant breeding is likely not, even though techniques such as radiation mutagenesis may be applied [which, though no more risky than other conventional breeding, may convey a sense of unnaturalness to the informed public].

The skepticism about novel plant breeding technologies is in line with consumer views in other steps of the food chain. For example Cardello et al.,^69^showed that irradiation and genetic modifications were associated with higher perceived risk and resulted in the greatest negative effects on likely use of foods. As pointed out by Siegrist,^70^ perceived risk, perceived benefit and perceived naturalness are most important for the acceptance of novel technologies. Mohorčich & Reese,^71^claim that lack of consumer-perceived GMO qualities is a consequence of a low consumer focus within agricultural GM technology, in favor of a focus on cost decrease and yield increase, resulting in qualities that are less apparent to the consumer.

Novel technologies may thus lower consumer trust in food. This poses a challenge since, in a European perspective, consumer trust in the food system is generally not very high. According to the EIT Food Trust Report ^72^ 55% of respondents representing European consumers consider that food products are generally safe, whereas 22% consider that they are not. The highest consumer trust is in farmers, with 67% of respondents indicating that they trust them, but less than 50% trust authorities and manufacturers.^72^ In a Swedish perspective, however, consumer trust in the food system is generally high, with 87% indicating strong trust in the Swedish food system, compared with 75% only five years earlier.^73^ Thus, increasing consumer trust in the food system and in novel techniques applied in the agrifood sector is a key issue.

Previous studies have shown that, compared with men, women are more negative to GM technology,^74,75^ less likely to accept GM foods or GM technology^22,76^ and perceive lower benefits from GMO.^77^ Women also perceive GM technology as a less moral method of agricultural production^78^. Gaskell et al.,^79^found that women tend to show less support for science and technology in general, indicating a gender gap.Lyndhurst,^80^ concluded that, compared with men, women are more concerned, less positive and see less benefit with food technologies. The close link between technology per se and plant breeding and GM technology can be expected to result in a gender difference in attitudes. The strong technology associations with both concepts point to low congruence between these two techniques and naturalness. Since women show higher preferences for natural food,^81^ it could be assumed that this leads to a less positive attitude not only to GMO, but also to conventional plant breeding, among women.

The impact of age on attitudes to GMO was explored by Mallinson et al.,^22^ who found that young adults are more accepting of GM foods. Greater concern about GMO among elderly consumers has been reported by Gaskell et al.^79^ Differences due to age as regards technology acceptance were also identified by Lyndhurst,^80^ who concluded that older persons are more likely to be concerned about novel food technologies in general.

### 2.1 Schwartz Value Theory

**Figure 1.**
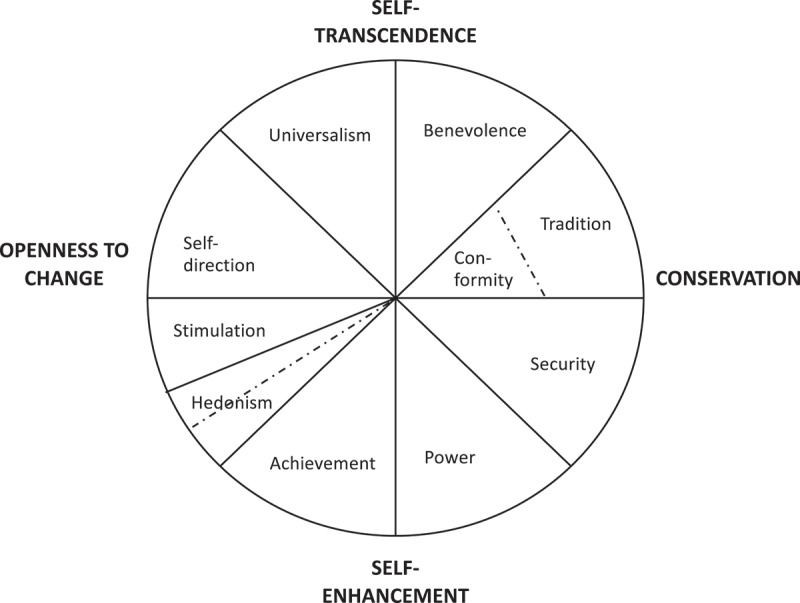
Illustration of components in Schwartz value theory as a circumplex containing 10 value classes, organized into four main value domains in a two-dimensional space 37

In order to explore values in the present study, Schwartz value theory was applied.^37,82,83^ It is structured around 10 distinctively different underlying values (see Table 1 for definitions and Fig. 1 for illustration). These values were measured here using the human values scale (HVS) for the European Social Survey [ESS) developed by Schwartz et al.^85^ The HVS consists of 21 short verbal portraits (adjusted for gender], to which the respondent is requested to indicate resemblance to oneself (see Appendix 1 for included questions and methods section for details on calculations). Each value is measured using 2–3 questions (Appendix 1 lists each question and the value it measures). Schwartz posits that these values are shared globally and guide humans throughout their life and in their daily living. Depending on individual differences in the weakness or strength of each value, individuals make different choices and show different degrees of openness to new situations, products or people.

**Table 1.** Definition of value types, a taxonomy of human values^84.^

Value	Definition
Self-direction	Independent thought and action (choosing, creating, exploring). Creativity, freedom, independent, curious, choosing own goals.
Stimulation	Excitement, novelty and challenge in life. Daring, a varied life, an exciting life
Hedonism	Pleasure and sensuous gratification for oneself. Pleasure, enjoying life.
Achievement	Personal success through demonstrating competence according to social standards. Successful, capable, ambitious, influential.
Power	Social status and prestige, control or dominance over people and resources. Social power, authority, wealth.
Security	Safety, harmony and stability of society, relationships and self. Family security, national security, social order, clean, reciprocation of favors.
Conformity	Restraint of actions, inclinations and impulses likely to upset or harm others and violate societal expectations or norms. Self-discipline, obedient, politeness, honoring parents and elders.
Tradition	Respect, commitment and acceptance of the customs and ideas that traditional culture or religion provide. Accepting one´s position in life, humble, devout, respect for tradition, moderate.
Benevolence	Preservation and enhancement of the welfare of people with whom one is in frequent personal contact. Helpful, honest, forgiving, loyal, responsible.
Universalism	Understanding, appreciation, tolerance and protection for the welfare of all people and for nature. Broadminded, wisdom, social justice equality, a world at peace, unity with nature, protecting the environment.

Schwartz theory concerns a system structured around dynamic relations between the values included (see Fig. 1). Some can be described as conflicting values (e.g. ‘benevolence’ and ‘power’), whereas others are more congruent, such as ‘conformity’ and ‘security’. According to Schwartz theory, the structure of the values is based on these relations of conflicts and consistency between values, which can be summarized in two orthogonal dimensions. The first (vertical), ‘self-enhancement’ versus ‘self-transcendence’, contrasts the values ‘achievement’ and ‘power’ (personal success and status) with the values ‘benevolence’ and ‘universalism’ (welfare of people and protection of nature). The second (horizontal dimension) covers ‘openness to change – conservation’, contrasting the values ‘stimulation’ and ‘self-direction’ (excitement in life and independency) with the values ‘tradition’, ‘conformity’ and ‘security’ (self-discipline, respect and security). As can be seen in Fig. 1, ‘hedonism’ (pleasure and enjoying life) is connected to both ‘openness to change’ and ‘self enhancement’.^37^

### 22. Influence of Values on Attitudes to GMO and Conventional Plant Breeding

The importance of values as determinants of consumer attitudes to GM foods has been raised in several studies,^23,86^ but no study has explored the corresponding patterns for conventional plant breeding. In studies examining the relationship between attitudes to GMO and values in accordance with Schwartz value theory,^37^ attitudes to GMO have been shown to be positively influenced by ‘power’ (self-enhancement)^46^ and negatively influenced by values related to responsibility for nature and the welfare of others (self-transcendence values.^87,88,89^ Whittingham et al.,^90^ identified a similar pattern in a study examining how values affect the perceived safety of GM food. This suggests that consumers who are positive to GMO are guided by the value ‘power’, which is defined by markers such as social status and prestige, control and dominance over people and resources. Dreezen et al. ^46^interpreted this as indicating a positive attitude to dominance, in this case dominance of humans over the natural environment. Following Schwartz value theory regarding the dynamic and even conflicting relations between opposing values, the opposing value [‘benevolence’) should apply to those who are negative to GMO. Benevolence is explained by Schwartz,^37^s a value defined by preservation and enhancement of the welfare of people with whom one is in frequent personal contact. The assumption that attitudes to GMO are negatively linked to benevolence is supported by studies exploring the link between negative attitudes to GMO and fear of long-term health effects of consuming GM foods ^38,91^ and studies showing that perceived high risk of GM foods leads to reduced acceptance, negative attitudes and negative emotional responses.^28,29,16,30,31^ Schwartz claims that values can be clustered due to consistency between values, which in this case would mean power and achievement belonging to the cluster ‘self-enhancement’ (personal success and status). ‘Self-enhancement’ is contrasted with universalism and benevolence (welfare of people and nature), i.e. representing values of ‘self-transcendence’ (see Fig. 1). Universalism has been defined as understanding, appreciation, tolerance and protection for all people and for nature.^37^ However, GMO has been identified as being associated with manipulation and unnaturalness,^20,79,92^ and consumer concern about nature has been identified as an antecedent of fear of GM crops.^30^ Thus it is reasonable to assume that attitudes to GMO are negatively linked to universalism. The importance of elucidating the concept of naturalness is also highlighted by Frewer et al.,^9^ who found that the debate and concerns around GMO often involve descriptions such as ‘unnaturalness’. This provides further support for the suggested link between attitudes to GMO and the value ‘universalism’. Based on the built-in dynamic in Schwartz value theory, achievement is the contrasting value to universalism (see Fig. 1). Therefore, individuals who are positive to GMO should be linked to achievement, defined as personal success through demonstrating competence according to social standards (self-enhancement).

Even though products resulting from conventional plant breeding can be expected to be considered less technology-intensive than GMO,^65^ it is reasonable to assume that the value structure explaining attitudes to conventional plant breeding is similar to that presented above for GMO (e.g. humans dominating over nature, associations to naturalness).

This led to the following hypotheses:

H1: *Consumer attitudes to conventional plant breeding are negatively influenced by universalism and benevolence, and positively influenced by power and achievement.*

H2: *Consumer attitudes to GMO are negatively influenced by universalism and benevolence, and positively influenced by power and achievement.*

## 3. Materials and Methods

Data were collected using a questionnaire (consumer panel, PFM Research in Sweden AB) completed on-line by 1500 Swedish consumers (750 male, 750 female) in June 2019. In selection of respondents, measures were taken to ensure an even gender distribution, equal age categories and representative distribution across the country and between urban areas and sparsely populated areas. All statistical analyses were conducted using IBM (SPSS, ver. 26). The sample for the survey was limited to consumers who lived in their own household and bought fruit and vegetables, to ensure that they were involved in handling vegetables.

Implementation of the survey followed the Swedish University of Agricultural Sciences policy for processing of personal data (https://www.slu.se/en/about-slu/contact-slu/personal-data/). The data were collected by PFM Research in Sweden AB and coded prior to delivery, ensuring anonymity. National and international agreements were followed. No reason for applying for ethical vetting from the Central Ethical Review Board (Etikprövningsnämnden) was identified, since questions on individual health per se were not included in the questionnaire. The general international code and guidelines on market and social research used by the International Chamber of Commerce^93^ were followed.

All respondents were asked the same questions, in the same order, and were requested to answer the questions in relation to the products fruit and vegetables. To ensure a common understanding of the concepts of conventional plant breeding and GMO, the following explanatory text was first presented to the respondents: (conventional plant breeding) “*Plant breeding is the change that humans have made, and are making, in plants to adapt them to our needs. The adaptation may be that the plant should yield many and large fruits or seeds, be easy to harvest, tolerate frost and drought, contain low levels of harmful or tasteless substances*” and (GMO) “*GMO is an abbreviation of genetically modified organism. [… It is an organism where the genetic material has been altered in a way that does not occur naturally through e.g. mating or cross-fertilization. … In a GMO, one or more DNA sequences have been added or removed*.” These explanations were based on previous definitions in conventional plant breeding^94^ and a definition of GMO provided by the Swedish Board of Agriculture.^95^

Using GM as a proxy for the whole range and great diversity of technologies available in genetic engineering may seem over-simplistic. However, GM has been confirmed as a term used by consumers seeking information on GM-related issues.^96^ Similarly, using the term “conventional plant breeding” may seem over-simplistic, considering the range of different methods that have been developed in the past 100–120 years. However, consumers are rarely aware of the differences between these, whereas the dichotomy between GM and conventional is determined by law and often perceived by consumers as a tangible distinction.^65^ The possibility of measuring participants´ understanding of the studied concepts was low, a limitation in the study, but the explanatory texts presented above were included with the hope of reducing misunderstandings.

The questionnaire covered three different aspects (see Appendix 1 for questions):

*1) Sociodemographic data*. Gender, age; divided into four groups: 25–34 yrs (group 1), 35–49 yrs (group 2), 50–64 yrs (group 3) and 65 yrs (group 4) and highest completed education, see Table 2. Questions were developed in communication with PFM Research in Sweden. Comparing the study sample and the Swedish population at large, the age groups were in line with the population in general, but the gender distribution and education level deviated somewhat (Table 2).

**Table 2.** Respondent sample and Swedish demographics (N = 1500)

Variable	Description	Sample	Swedish population^a^
**Gender**	Female	47.9%	49.7%
	Male	52.1%	50.3%
**Age**	25–34	19%	20%
	35–49	27%	27%
	50–64	26%	25%
	>65 yrs	28%	28%
**Education**	Elementary school	6.1%	11%
	Gymnasium	38.4%	45%
	University	55.5%	44%

^a^. 97

*2) Attitudes*. The question asked was: ‘What is your attitude to the concept of plant breeding described as ‘conventional´ and ‘GMO’? Respondents were asked to indicate their response using a 7-point Likert scale (1 = completely negative to 7 = completely positive) or 8 = don´t know. Questions formulated by the authors.

*3) Values*. Values were measured using the human values scale (HVS) for the European Social Survey [ESS) developed by Schwartz et al.^85^ The HVS consists of 21 short verbal portraits (adjusted for gender], to which the respondent is requested to indicate resemblance to oneself (see Appendix 1 for complete list of questions A-U). Response was measured using a 6-point Likert scale (1 = very much like me to 6 = not like me at all) or 7 = don´t know. Each value is measured using 2–3 questions, Appendix 1 lists the values that each question measures. Since the HVS questions are adjusted for gender, the on-line questionnaire was designed to ensure that each participant received the right questions due to gender. Before computing the mean scores for the 10 values, items were inverted, so that higher scores represented greater value importance. Internal reliabilities calculated for the scales showed that the alpha values for the 10 values ranged between .26 (tradition) and .76 [achievement). Although ‘tradition’ had low reliability, according to,^98^even values with low reliability can provide substantial predictive and discriminant validity. Following recommendations by Schwartz,^98^and Schwartz et al. ^85^ values were centered prior to further analysis, in order to make corrections for individual differences. Means were thus calculated for all 10 values, each individual´s mean score over all 21 value items was computed and values were centered.

## 4. Results

Replies from respondents indicating “don’t know” for questions relating to attitudes and values were excluded from the results. All results are presented separately for GMO and conventional plant breeding. Calculations were made for the four different age groups (1–4]. When exploring values, attitudes to plant breeding and GMO were separated into three attitudinal groups: 1) negative (mean value scores 1–2); 2) neutral (3–5); and 3) positive (6–7).

### 4.1 Differences in Attitudes to Plant Breeding and GMO and Differences Due to Age and Gender

In order to explore differences in attitudes to conventional plant breeding and GMO, a paired-samples t-test was conducted. The outcome revealed a significantly more positive attitude to conventional plant breeding (4.83±1.54) than to GMO (3.44±1.87) (t (1184) = 32.72, p<.000 (two-tailed)). The mean decrease in attitude was 1.39 (95% confidence interval (CI) = 1.37, 1.47). The ŋ^2^ value was .47, indicating large effect size.^99^

To investigate gender-related differences in attitudes to plant breeding, an independent samples t-test was conducted. The results revealed that men expressed a significantly more positive attitude to conventional plant-breeding (5.12±1.54) compared with women (4.52±1.49) (*t* (1183) = 6.84, *p* = .000 (two-tailed), ^2^ =ŋ^2^ .04), indicating an almost moderate effect.

**Figure 2.**
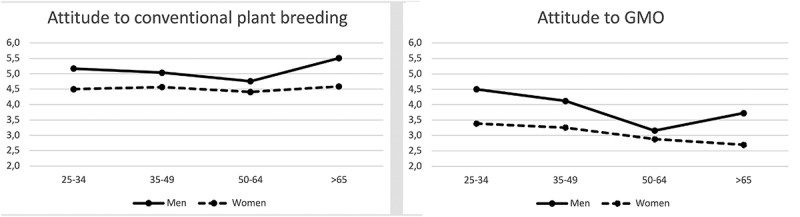
Attitudes to (left) conventional plant breeding and (right) genetically modified organisms (GMO), divided by gender and age groups

In order to further explore the impact of gender and age, two-way ANOVA was conducted. No statistically significant interaction effect between gender and age was found (*F*(3, 1177) = 2.22, *p* = .08). The calculations showed a statistically significant main effect for gender (*F*(1,1177) = 45.20, *p* = .000), but with quite small effect size (ŋ^2^=.04). They also showed a statistically significant main effect for age (*F*(3,1177) = 5.21, *p* = .001), but with small effect size (=ŋ^2^ .01) (Fig. 2).

As can be seen from Fig. 2, men in all age categories expressed a more positive attitude to conventional plant breeding, compared with women in the same age group, which is in line with the significant main effect identified for gender. Differences between age groups were less pronounced, but a significant main effect for age was identified. Post-hoc comparison using the Tukey HSD test revealed a significant difference at *p<*.05 only between age groups 3 and 4, with the older of these age groups (>65 yrs) expressing a significantly more positive attitude to conventional plant breeding compared with the younger group (50–64 yrs).

**Figure 3.**
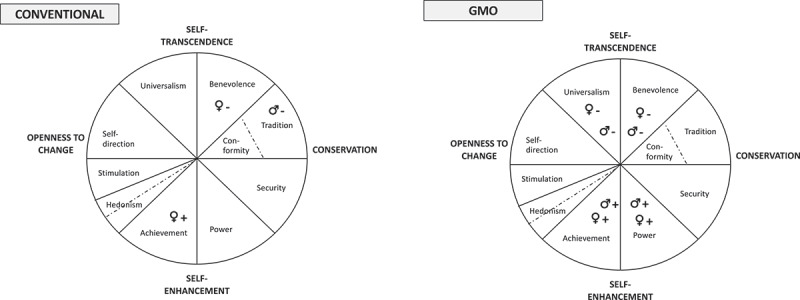
Correlation of attitudes to: (left diagram) conventional breeding and (right diagram) genetically modified organisms (GMO) with Schwartz value theory components among male (**♂**) and female (**♀**) respondents. “+” indicates a positive correlation and “–” a negative correlation

The calculations for attitudes to GMO revealed an interaction effect between gender and age that was statistically significant (*F*(3, 1177) = 3.26, *p* = .021). Therefore, additional calculations were made for gender and age separately (Fig. 3).

An independent samples t-test revealed a significantly more positive attitude to GMO among men (3.84±1.95) than among women (3.01±1.69) (*t* (1183) = 7.80, *p* =.000, two-tailed.  ŋ^2^= .05), indicating an almost moderate effect.

Differences between different age groups were explored through one-way between-group ANOVA. The results indicated a statistically significant difference at the *p<*.001 level (*F* (3,1181) = 15.93, *p* = .000). Despite being statistically significant, the actual difference in mean scores between the groups was quite small (ŋ^2^= .04). On conducting a post-hoc comparison using the Tukey HSD test, a significant difference at *p<*.05 was identified between age groups 1 and 2 relative to age groups 3 and 4. The two youngest age groups expressed significantly more positive attitudes to GMO than the two oldest age groups (Fig. 2).

### 4.2 Differences in Attitudes to Conventional Plant Breeding Due to Values

To test hypothesis H1 that attitudes to conventional plant breeding are negatively influenced by universalism and benevolence and positively influenced by power and achievement, an introductory explorative correlation analysis was made for attitude to conventional plant breeding and all 10 values (Table 3). Within the male group, a significant negative correlation was found for the value ‘tradition’. Within the female group, significant correlations were identified for ‘benevolence (neg.)’ and ‘achievement (pos.)’, see Fig. 3.

**Table 3.** Correlations between different values and consumer attitudes to conventional plant breeding, shown separately for men and women

Value	Men	Women
Conformity	.051	.042
Tradition	−.086*	−.026
Benevolence	−.017	−.139**
Universalism	−.049	−.051
Self-direction	.037	−.012
Stimulation	−.058	.016
Hedonism	.014	−.018
Achievement	.074	.085*
Power	.011	.052
Security	.035	.025

^*^p<.05 (2-tailed), **p.01 (2-tailed), investigated using Pearson product-moment correlation coefficient.

Values were also explored with regard to gender and three different attitudinal groups (negative, neutral, positive). One-way between-group ANOVA was conducted to examine differences in expressed benevolence, universalism, achievement and power between these three groups for conventional plant breeding. The value ‘tradition’ was also examined, considering the correlation identified for this variable (see Table 3).

Calculations for the male group showed statistically significant differences for ‘tradition’ (*F*(2,614) = 4.37, *p* = .013) between the neutral and positive groups (Table 4). As expected, no statistically significant difference was found for benevolence, universalism, achievement or power between the three attitudinal groups.

**Table 4.** Measures (centered) of the values tradition, benevolence, universalism, achievement and power, separated by gender and attitudes to conventional plant breeding (negative, neutral and positive)

Value		Negative attitude		Neutral attitude		Positive attitude		**(ŋ^2^)**
	N(%)	M = 38(6%) W = 59(10%)		M = 298(48%) W = 356(63%)		M = 281 (46%) W = 153(27%)		
**Tradition**	Men	−.12.±.75^ab^		.06±.85^a^		−.16±.93^b^		.01
	Women	−.10±1,18		−.11±.88		−.20±.97		-
**Benevolence**	Men	.99±.74		.75±.70		.79±.75		-
	Women	1.33±.67^a^		1.08±.66 ^b^		1.01±.76^b^		-
**Universalism**	*Men*	.85±.85		.54±.74		.54±.88		-
	Women	1.10±.85		.87±.78		.95±.77		-
**Achievement**	*Men*	−1.01± 1.10		− .80±.91		−.69±1.00		-
	Women	−1.24±.98^a^		−1.02±.96^ab^		−.88±1,04^b^		-
**Power**	Men	−.90±.76		−.79±.81		−.82±.81		-
	Women	−1.27±.79		−1.04±.69		−1.13±.79		-

^ab^Different letters indicate significant difference between the three attitudinal groups for M = men and W = women. Values shown are mean±standard deviation.

For women, the results showed significant differences between attitudinal groups for ‘benevolence’ (F(2,565) = 4.78, *p* = .009), with the negative group showing significantly higher benevolence than the other two groups (Table 4). Measures for ‘achievement’ (F(2,565) = 2.93, *p* = .05) revealed that the negative group expressed numerically lower values of achievement compared with the positive group, but the differences were not significant. No significant differences were found for tradition, universalism or power.

The results for men did not support H1: *Consumer attitudes to plant breeding are negatively influenced by universalism and benevolence, and positively influenced by power and achievement.*

The results for women supported H1 for benevolence and achievement, but not for universalism and power.

### 4.3 Differences in Attitudes to GMO Due to Values

To test H2, correlational analysis was first performed for attitude to GMO and all 10 values considered (Table 5). Significant correlations (both men and women) were found for benevolence, universalism, achievement and power (Fig. 3).

**Table 5.** Correlations between different values and attitudes to genetically modified organisms (GMO), shown separately for men and women

Values	Men	Women
Conformity	.023	−.039
Tradition	−.063	−.072
Benevolence	−.091*	−.111**
Universalism	−.106**	−.158**
Self-direction	−.002	.023
Stimulation	.003	.043
Hedonism	.003	.062
Achievement	.111**	.167**
Power	.127**	.169**
Security	.017	−.034

^*^p<.05 (2-tailed), **p<.01 (2-tailed), investigated using Pearson product-moment correlation coefficient.

Values were explored with regard to gender and the three different attitudinal categories (negative, neutral, positive). One-way between-groups ANOVA was conducted to examine differences in expressed benevolence, universalism, achievement and power between these three groups (Table 6).

**Table 6.** Measures (centered) of the values tradition, benevolence, universalism, achievement and power, separated by gender and attitudes (negative, neutral and positive) to genetically modified organisms (GMO)

Value		Negative attitude		Neutral attitude		Positive attitude		(ŋ^2^)
	N (%)	M = 192(31%) W = 260(46%)		M = 267(43%) W = 256(45%)		M = 158(26%) W = 52(9%)		
**Benevolence**	Men	.93±.70^a^		.68±.70^b^		.77±.79^ab^		.02
	Women	1.12±.67		1.08±.68		.92±.68		-
**Universalism**	Men	.76±.81^a^		.49±.72^b^		.47±.94^ab^		.01
	Women	1.04±.76^a^		.80±.77^b^		.86±.89^ab^		.02
**Achievement**	Men	−.92±.95^a^		−.70±.93^ab^		.67±1.04^b^		.01
	Women	−1.14±.96^a^		−.93±.97^b^		−.73±1.18^b^		.02
**Power**	Men	−.95±.79^a^		−.76±.76^b^		−.71±.87^b^		.01
	Women	−1.18±.73^a^		−1.04±.69^ab^		−.87±.88^b^		.02

^a^Different letters indicate significant difference between the three attitudinal groups for M = men and W = women. Values shown are meanstandard deviation.

Calculations made on the male group showed statistically significant differences between the three attitudinal groups for benevolence (*F*(2,614) = 3.4, *p* = .001), universalism (*F*(2,614) = 3.68, *p* = .026), achievement (*F*(2,614) = 3.75, *p* = .024) and power (*F*(2,614) = 4.62, *p* = .010). For women, the results showed statistically significant differences between the three attitudinal groups for universalism (*F*(2,565) = 6.57, *p* = .002), achievement (*F*(2,565) = 5.13, *p* = .006) and power (*F*(2,565) = 5.22, *p* = .006). Men and women expressing negative attitudes to GMO both self-reported significantly higher levels of universalism, but for benevolence a significant result was only found among men. Within the positive group, significantly higher levels of achievement and power were self-reported by both men and women.

For men and women, the results support H2: *Consumer attitudes to GMO are negatively influenced by universalism and benevolence, and positively influenced by power and achievement.*

## 5. Discussion

This study examined links between values identified in accordance with Schwartz value theory^37^ and positive and negative attitudes to GMO and conventional plant breeding. It also compared differences in attitudes to GMO and conventional plant breeding among consumers in general, and in relation to gender and age.

The results showed that consumers expressed more positive attitudes to conventional plant breeding than to GMO, and that men expressed more positive attitudes than women to both conventional plant breeding and GMO. However, younger consumers did not express a more positive attitude to conventional plant breeding compared with older consumers. Surprisingly, for men the oldest consumer group (>65 yrs) expressed the most positive attitude to conventional plant breeding. For GMO, younger age groups had a more positive attitude than older groups.

For men, no correlations were found for conventional plant breeding and universalism, benevolence, power or achievement. However, the results revealed a correlation for the value tradition (significant difference between the positive and negative group) (H1). Corresponding results for women revealed correlations for benevolence and achievement, but not universalism and power. With regard to GMO, correlations were found supporting the assumption that (for both men and women) attitudes to GMO were negatively influenced by universalism and benevolence, and positively influenced by power and achievement (H2).

The outcome that consumers in general have a less positive attitude to GMO than to conventional plant breeding is well in line with previous findings.^17,65–67,100^ As proposed by Siegrist,^70^ this may point at no perceived benefits with the technology, and also perceived risks.^69^ A surprising finding was that the attitude to conventional plant breeding (4.83, on a scale from 1–7) was within the neutral range, especially considering that all fruit and vegetables that can be bought in food stores come from crops that have been developed through breeding in modern times. A reason for this may be that consumers generally have low awareness of the overall breeding process.^65^ But nevertheless it is intriguing considering that Swedish consumers show high trust in the food system and perceive less risks than their European counterparts.^72,73^ There are a number of possible explanatory variables for the identified neutral, rather than positive, attitude to conventional plant breeding. Skepticism about the use of technology in general could be one important explanatory variable, e.g. Inglehart,^101^showed that reluctance to accept technology and innovation can be seen as a marker of a desire to replace economic growth with concern for the environment, personal development and civil liberties. This goes against the development advocated by OECD,^102^ which recognizes a need for technical innovations in particular to develop new sustainable solutions to identified problems. Other explanations could be lack of knowledge concerning the link between conventional plant breeding per se and the products available in supermarkets, in line with findings presented by van Heuvel et al.^65^

The gender effect revealed in Fig. 2 confirms previous findings that men express a more positive attitude to GMO compared with women.^22,74,75^ It also shows that this gender difference applies for conventional plant breeding. Differences between the genders were further revealed on separating attitudes into three attitudinal groups (positive, neutral, negative) concerning conventional plant breeding and GMO (see Tables 4 and 6).

The lack of evidence that younger consumers are more positive to conventional plant breeding is interesting. As illustrated in Fig. 2, the oldest male group had the most positive attitude to conventional plant breeding. One explanation for this finding could be that the plant breeding industry was most prominent and successful in Sweden during the 1930s-1970s,^103^ during which period there were also significant public investments in plant breeding. It should also be noted that institutionalized plant breeding in the past has very likely been mainly a male occupation, and this may also contribute to a gender bias in the perception of these activities. Despite the fact that society as a whole cannot be regarded as particularly technology-hostile today compared with previous generations, plant breeding and food production may be a special case. Memories of food shortages that may persist among the older generation, either self-experienced or learned from parents, do not exist with the younger generation today, when food is taken for granted. Therefore, technological development in food production is not seen as desirable or even necessary. Technology has been a critical key factor in the development of society and, not least, in the development of food and fruit and vegetables. In recent decades, consumer trust in the food system has decreased,^104^ but Swedish consumers show high trust in the food system and perceive less risks than their European counterparts.^72,73^

A study by Tanaka,^105^identified trust as the most important factor in acceptance of conventional plant breeding. The results in the present study may thus be explained by generational differences in trust and in attitudes to food technology. Regarding attitude to GMO and age, the results, in particular for the female cohort in this study (Fig. 2), confirm findings in previous studies^22,74^ that younger consumers have a more positive attitude to GMO compared with older consumers. This implies a change in view on the use of GMOs. It may reflect generally higher technology optimism among the younger generation, in combination with factors discussed above for the older generation in relation to plant breeding in general. It may also be because Swedish consumers show high trust in the food system and perceive less risks than their European counterparts.^72,73^

It is clear that many factors play a role in public perceptions on plant breeding and related technologies. An intriguing finding in the present study was that men consistently, in all age categories, expressed a more positive attitude to conventional plant breeding and to GMO compared with women.

Comparisons of value structures, following Schwartz value theory, with attitudes to conventional plant breeding and GMO revealed no significant correlations for men between attitudes to conventional plant breeding and the values ‘universalism’, ‘benevolence’, ‘power’ and ‘achievement’ (see Table 3). The results for the male group revealed a surprising result for conventional plant breeding, with a significant negative correlation for the value ´tradition´, a concept described by terms such as respect, commitment and acceptance of the customs and ideas that conventional culture or religion provide. The negative correlation identified suggests that, among men, high scores on tradition (e.g. accepting one’s position in life, humble) indicate less positive attitudes to conventional plant breeding. This finding does not support hypothesis H1, which suggested universalism (e.g. unity with nature, protecting the environment) and benevolence (e.g. helpful, honest, forgiving). This finding adds new knowledge on the values that underlie attitudes to conventional plant breeding (among men). For women, the results showed a negative correlation between attitudes to plant breeding and ‘benevolence’ and a positive correlation for ‘achievement’. This suggests that female respondents who were in favor of conventional plant breeding valued personal success (achievement), whereas those who expressed negative attitudes valued enhancement of the welfare of people with whom they are in frequent personal contact (benevolence). The lack of correlation for ‘universalism’ and ‘power’ suggests that these values do not significantly explain women’s attitudes to plant breeding.

The finding, for both men and women, that attitudes to GMO are negatively influenced by universalism and benevolence, and positively influenced by power and achievement [H2), partially reinforces previous findings by Dreezen et al. ^46^ that ‘power’ may be linked to a positive attitude to GMO. However, more importantly, our findings point to a link between ‘universalism’, which Dreezen et al.,^46^did not identify. Bech-larsen & Grunert,^87^identified value patterns in line with our findings, but explored value structures at a more aggregated level. The studies by Saher et al. ,^89^,and Honkanen & Verplanken ^88^were designed to explore only negative attitudes. The values structure identified for consumers (separated by gender] who express positive and negative attitude to GMO adds to the current understanding that attitudes to GMO represent two polarized value structures, with positive attitudes correlated with self-enhancement (power and achievement) and negative attitudes with self-transcendence (universalism and benevolence).

In combination, the results showed that women had a significantly more negative attitude to conventional plant breeding compared with men. An explanation can be found in the correlation identified for the value ‘benevolence’. Following the definition of ‘benevolence’ (see Table 1), women who express negative attitudes may perceive risk to their family from consuming fruit and vegetables produced through conventional plant breeding. One additional explanation could be related to the fact that women do more of the grocery shopping and cooking than men, and hence are more in contact with fruit and vegetables. Technological advances may thus be more real and less abstract for women than for men, which in turn creates more threat (more negative attitudes) for women compared with men. However, it is worth mentioning that the female respondents who expressed positive attitudes were linked to the value ‘achievement’, defined as personal success through demonstrating competence according to social standards.

As in the case of negative attitudes to GMO, explanations can also be related to the concept of naturalness. For example Lampila et al.,^67^showed that mistrust of a product (fruit) increased when new properties (flavonoids) were introduced, as consumers perceived the product not to be sufficiently natural. This highlights an important area to be considered by plant breeders, namely choosing traits to develop while still preserving the natural image of the product. For consumers who value naturalness and use this as a safety marker, technological development, whether as GMO or conventional plant breeding, will adversely affect their attitude to both these technologies. The importance of listening to consumers is highlighted by Loizou et al.,^106^ who stress the importance of not unanimously focusing on the possibilities of technology. Frewer et al.,^107^suggest developing food technology through exploring psychological, social, political and historical issues, as consumers who experience control of their consumption can be expected to express higher consumer acceptance. Finally, the importance of including both farmers and consumers in the plant breeding process (conventional and GMO) in a participatory manner^108^ must be considered. According to Janick,^109^ breeding objectives for horticultural crops must be consumer-orientated, since consumers make individual decisions and choose between different cultivars. Therefore, unique quality traits, rather than yield per se, should be the guiding principle for breeding objectives for horticultural crops.

It is important to note that the present study involved only Swedish consumers. The results are not directly transferrable to other cultural contexts, especially since there are known to be great differences with regard to risks, benefits and moral considerations between continents and countries, e.g. between Europe and the USA,^16^ and between EU countries.^19^

## 6 Conclusions and Policy Implications

This study identified a number of issues that need further investigation to increase understanding of the importance of value structure for attitudes to both GMO and conventional plant breeding. The gender gap identified for GMO (and also for conventional plant breeding) and the lack of clear explanations for this create a need for further studies. Within the Swedish food system, consumers are generally unconnected with the plant breeding sector, which can lead to lower tolerance for not only GM technology, but also conventional plant breeding. The results revealed a rather large difference in relation to gender. Around a quarter of men (26%) expressed a positive attitude to GMO, compared with only 9% of participating women. The corresponding proportions for those with a negative attitude were more similar (31% and 36%, respectively). For conventional plant breeding, the results showed that less than half of all participating men (46%) and around a quarter (27%) of female respondents had a positive attitude, while 6% of male respondents and 10% of female respondents had a negative attitude, to conventional plant breeding. Since conventional plant breeding is essential to maintain, and also further develop, high-performing cultivars of fruit and vegetables under future climate conditions, these negative attitudes have serious connotations. Plant breeding techniques are key in developing the necessary innovations and solutions to produce food (including fruit and vegetable), and negative consumer perceptions of these techniques may prevent essential product and cultivation development within the food supply sector.

The results presented, especially those showing differences between consumers who are either positive or negative to GMO and conventional plant breeding add new knowledge for bridging this gap. Better knowledge of underlying explanatory variables, such as values, can provide valuable support in understanding why consumers express positive or negative attitudes to GMO and conventional plant breeding. The generational difference identified, with the oldest (male) consumer group expressing the highest positive attitude toward conventional plant breeding, is interesting. Through exploring this specific consumer group, an understanding of how and why this type of technological food development is not viewed as negative can be developed. Such knowledge can be important to plant breeders and the horticultural industry in general when developing and promoting products and processes that are perceived as relevant and trustworthy by consumers.

Future studies should explore value structures among consumers who perceive congruence between sustainability and technological development, in order to understand whether and how sustainability can be driven by technology-friendly consumers. Based on the findings presented in this paper, one way forward could be to explore young consumers, especially those expressing high levels of universalism and benevolence and who are also positive to technological development within food, e.g. GMO and similar technologies. This is especially important when considering the need for technological development within the food system.

## Appendix 1

Compilation of questions included in the questionnaire.

Questions 1–5 developed in dialogue with PFM Research in Sweden AB.

Questions 6–7 developed by authors

Questions A-U in the Human Value Scale developed by.^85^

**Table:**

Question	Answering options
1. What is your age?	24 years or younger, 25–34 years, 35–49 years, 50–64 years, 65 years or older
2. Are you … ?	Male, female
3. What is your postal code?	
4. What type of household do you live in?	Single-person household, Multi-person household with children, Multi-person household without children, Living at home with parents/not living in own household
5. What is your highest completed level of education?	Primary school/Equivalent, High school/Equivalent, University/College, none completed
6. What is your attitude to the concept of plant breeding described as ’conventional´? “*Plant breeding is the change that humans have made, and are making, in plants to adapt them to our needs. The adaptation may be that the plant should yield many and large fruits or seeds, be easy to harvest, tolerate frost and drought, contain low levels of harmful or tasteless substances*”	(1 = completely negative to 7 = completely positive] or 8 = don´t know.
7. What is your attitude to the concept of plant breeding described as `GMO`? *“It is an organism where the genetic material has been altered in a way that does not occur naturally through e.g. mating or cross-fertilization. … In a GMO, one or more DNA sequences have been added or removed*.”	(1 = completely negative to 7 = completely positive) or 8 = don´t know.

## Human Values Scale [ESS) by^85^

Divided for male and female respondents, with separate questionnaires for men and women. The abbreviation after the question shows the value that each question measures.

SD = Self-direction

ST = Stimulation

HE = Hedonism

AC = Achievement

PO = Power

SE = Security

CO = Conformity

TR = Tradition

BE = Benevolence

UN = Universalism

Instruction to respondent: Now I will briefly describe some people. Please listen to each description and tell me how much each person is or is not like you. Use this card for your answer.

Answering alternatives; Very much like me, Like me, Somewhat like me, A little like me, Not like me, Not like me at all and Don´t know.

A. Thinking up new ideas and being creative is important to him/her. He/she likes to do things in his/her own original way. SD

B. It is important to him/her to be rich. He/she wants to have a lot of money and expensive things. PO

C. He/she thinks it is important that every person in the world should be treated equally. He/she believes everyone should have equal opportunities in life. UN

D. It’s important to him/her to show his abilities. He/she wants people to admire what he/she does. AC

E. It is important to him/her to live in secure surroundings. He/she avoids anything that might endanger his/her safety. SE

F. He/she likes surprises and is always looking for new things to do. He/she thinks it is important to do lots of different things in life. ST

G. He/she believes that people should do what they’re told. He/she thinks people should follow rules at all times, even when no-one is watching. CO

H. It is important to him/her to listen to people who are different from him. Even when he/she disagrees with them, he/she still wants to understand them. UN

I. It is important to him/her to be humble and modest. He/she tries not to draw attention to himself. TR

J. Having a good time is important to him/her. He/she likes to “spoil” himself. HE

K. It is important to him/her to make his own decisions about what he/she does. He/she likes to be free and not depend on others. SD

L. It’s very important to him/her to help the people around him. He/she wants to care for their well-being. BE

M. Being very successful is important to him/her. He/she hopes people will recognize his achievements. AC

N. It is important to him/her that the government ensures his safety against all threats. He/she wants the state to be strong so it can defend its citizens. SE

O. He/she looks for adventures and likes to take risks. He/she wants to have an exciting

life. ST

P. It is important to him/her always to behave properly. He/she wants to avoid doing anything people would say is wrong. CO

Q. It is important to him/her to get respect from others. He/she wants people to do what he says. PO

R. It is important to him/her to be loyal to his friends. He/she wants to devote himself to people close to him/her. BE

S. He/she strongly believes that people should care for nature. Looking after the environment is important to him/her. UN

T. Tradition is important to him/her. He/she tries to follow the customs handed down by his/her religion or his/her family. TR

U. He/she seeks every chance he can to have fun. It is important to him/her to do things that give him/her pleasure. HE
